# Functional Characterization of SMG7 Paralogs in *Arabidopsis thaliana*

**DOI:** 10.3389/fpls.2018.01602

**Published:** 2018-11-06

**Authors:** Claudio Capitao, Neha Shukla, Aneta Wandrolova, Ortrun Mittelsten Scheid, Karel Riha

**Affiliations:** ^1^Gregor Mendel Institute of Molecular Plant Biology, Austrian Academy of Sciences, Vienna Biocenter, Vienna, Austria; ^2^Central European Institute of Technology, Masaryk University, Brno, Czechia

**Keywords:** nonsense mediated RNA decay, meiosis, SMG7, gene duplication, Arabidopsis

## Abstract

SMG7 proteins are evolutionary conserved across eukaryotes and primarily known for their function in nonsense mediated RNA decay (NMD). In contrast to other NMD factors, SMG7 proteins underwent independent expansions during evolution indicating their propensity to adopt novel functions. Here we characterized SMG7 and SMG7-like (SMG7L) paralogs in *Arabidopsis thaliana*. SMG7 retained its role in NMD and additionally appears to have acquired another function in meiosis. We inactivated SMG7 by CRISPR/Cas9 mutagenesis and showed that, in contrast to our previous report, SMG7 is not an essential gene in Arabidopsis. Furthermore, our data indicate that the N-terminal phosphoserine-binding domain is required for both NMD and meiosis. Phenotypic analysis of SMG7 and SMG7L double mutants did not indicate any functional redundancy between the two genes, suggesting neofunctionalization of SMG7L. Finally, protein sequence comparison together with a phenotyping of T-DNA insertion mutants identified several conserved regions specific for SMG7 that may underlie its role in NMD and meiosis. This information provides a framework for deciphering the non-canonical functions of SMG7-family proteins.

## Introduction

In eukaryotic cells, gene expression is controlled by several surveillance mechanisms that assure accurate and robust production of functional proteins. One such control mechanism is nonsense mediated RNA decay (NMD), which degrades aberrant RNAs (He and Jacobson, 2015; Lykke-Andersen and Jensen, 2015; Hug et al., 2016; Raxwal and Riha, 2016). NMD typically targets transcripts containing premature termination codons (PTC) that arise as a consequence of a missense mutation or alternative splicing, leading to the translation of a truncated protein. NMD is an evolutionary highly conserved pathway that may have co-evolved with the acquisition of introns (Farlow et al., 2010). Although NMD is one of the most well-studied RNA surveillance pathways, its exact molecular mechanism is not yet fully resolved. The consensus model, based on studies across a range of organisms, suggests that NMD is induced by the premature translation termination at stop codons in the absence of a full complement of canonical translation termination signals (He and Jacobson, 2015; Lykke-Andersen and Jensen, 2015).

The most detailed insights into the NMD mechanism were obtained in mammals (He and Jacobson, 2015; Lykke-Andersen and Jensen, 2015; Hug et al., 2016). The key step is the phosphorylation and activation of the RNA helicase UPF1 by the SMG1 kinase in response to a ribosome stalled at a PTC. Mammalian NMD primarily targets transcripts harboring introns in their 3′UTR and UPF1 activation is facilitated by the presence of an exon–exon junction 50–55 nucleotides downstream of the PTC. This is sensed via interaction with the exon junction complex, and is mediated by UPF3B and UPF2. Once phosphorylated, UPF1 is recognized by the phosphoserine-binding proteins SMG5, SMG6, and SMG7 that induce degradation of the aberrant mRNA. SMG5, SMG6, and SMG7 belong to the same protein family, which is characterized by a phosphoserine-binding domain that structurally resembles 14-3-3 proteins (Fukuhara et al., 2005). Their association with UPF1 defines two major RNA degradation pathways. SMG6 itself acts as an endonuclease cleaving mRNA in the vicinity of the PTC. SMG5 and SMG7 bind phosphorylated UPF1 as a heterodimer (Jonas et al., 2013; Nicholson et al., 2018). SMG7 recruits the CCR4-NOT deadenylase complex and induces deadenylation-dependent decapping, which is followed by 5′-3′mRNA degradation (Unterholzner and Izaurralde, 2004; Loh et al., 2013). NMD in human cells mainly relies on SMG6 mediated degradation, while the SMG5-SMG7 dimer seems to act as a back-up pathway.

The NMD mechanism described in mammals appears to be conserved in plants, although some aspects differ (Kerenyi et al., 2008; Shaul, 2015). NMD in plants acts efficiently on transcripts containing 3′UTR introns, but also on transcripts that possess long 3′UTR without introns (Kertesz et al., 2006; Kurihara et al., 2009; Kalyna et al., 2012; Drechsel et al., 2013; Degtiar et al., 2015). This mechanism, known as the faux 3′UTR model, was described in budding yeast and postulates that the long distance between the PTC and poly(A)-tail fails to provide the proper context for translation termination and sensitizes transcripts to NMD (He and Jacobson, 2015; Hug et al., 2016). Functional studies in tobacco showed that both NMD pathways require UPF1, UPF2, and SMG7, while components of the exon junction complex are only involved in the intron-based mechanism (Kerenyi et al., 2008). As in mammals, phosphorylation of UPF1 is critical for late steps of plant NMD (Merai et al., 2012; Kerenyi et al., 2013). The SMG1 kinase is present in the majority of plants, but has been repeatedly lost in the *Brassicaceae* family, suggesting that its function can be substituted by an as yet unknown kinase (Lloyd and Davies, 2013; Causier et al., 2017). Phosphorylated UPF1 recruits SMG7 through its terminal 14-3-3 domain, which in turn mediates RNA degradation and UPF1 relocalization to P-bodies (Merai et al., 2012). Plants do not possess SMG5 and SMG6 paralogs, but the genomes of dicots encode an additional SMG7-like (SMG7L) protein (Riehs et al., 2008). Nevertheless, functional studies in grapevine indicate that SMG7L has lost its NMD activity (Benkovics et al., 2011).

Downregulation of NMD in Arabidopsis leads to a strong immune response that is caused by derepression of TIR-NB-LRR immune receptors (Jeong et al., 2011; Rayson et al., 2012; Riehs-Kearnan et al., 2012; Gloggnitzer et al., 2014; Raxwal and Riha, 2016). UPF1 and SMG7 are understood to act in subsequent steps of a linear pathway; therefore, their inactivation should have similar biological effects. However, the phenotypes of UPF1 and SMG7 in Arabidopsis differ substantially. A null *upf1-3* mutation causes seedling lethality due to massive activation of the immune response (Yoine et al., 2006; Riehs-Kearnan et al., 2012). This lethality can be rescued by the inactivation of PAD4, a key component of pathogen signaling, but the *upf1-3 pad4* double mutants still exhibit retarded growth and pleiotropic developmental defects. Analysis of plants carrying different T-DNA insertions in the SMG7 gene showed range of different phenotypes. The most N-terminal disruption in the 14-3-3 domain (*smg7-5*) was proposed to cause embryonic lethality, as no mutant plants could be recovered (Riehs et al., 2008). *Smg7-1* and *smg7-3* mutants carrying insertions in the central region of SMG7 are viable, but exhibit retarded growth caused by pathogen response activation and are infertile due to abortive meiosis (Riehs et al., 2008; Bulankova et al., 2010). While the vegetative growth defects are fully rescued in *smg7-1 pad4* double mutants, meiosis is still defective. Furthermore, the meiotic defects are not observed in *upf1-3 pad4* mutants, suggesting that the role of SMG7 in meiosis is not linked to NMD (Riehs-Kearnan et al., 2012).

Here, we aimed to clarify whether Arabidopsis SMG7 is an essential gene by characterizing SMG7-loss of function mutants generated by CRISPR-Cas9 mutagenesis. Furthermore, we investigated the requirements for the 14-3-3 domain in meiosis and the redundancy between SMG7 and SMG7L paralogs. Finally, we used phylogenetic analysis to search for motifs that may define SMG7 function in NMD and meiosis.

## Materials and Methods

### Plant Material

Seeds of *Arabidopsis thaliana* were grown in soil in phytotrons at 20–22°C with a photoperiod of 16 h light/8 h dark. All wild type and mutant plants were derived from *A. thaliana* ecotype Col-0. The *smg7-1* and *smg7-6* mutants used in this study were characterized previously (Riehs et al., 2008; Riehs-Kearnan et al., 2012) and the *smg7l-1* mutation was obtained from the Arabidopsis Stock Centre (SAIL_634H06).

### Vectors and Transgenic Plants

We used the CRISPR/Cas9 system developed for targeted mutagenesis in plants (Fauser et al., 2014). The CRISPR sequence guiding the Cas9 nuclease was designed to target the second exon of the SMG7 gene downstream of and near to the ATG codon. Briefly, the guide RNA was created by cloning the duplex oligonucleotide 5′-CTTGGCTCGCTCCCATGAAG-3′ into the *Bbs*I site of pEN-Chimera (Fauser et al., 2014). The region coding for the gRNA was transferred into pDe-CAS9 using the Gateway^TM^ cloning system, creating the binary vector pCCC40. Transgenic plants were generated by Agrobacterium-mediated floral-dip transformation of *A. thaliana* ecotype Col-0. Transgenic plants were screened in the T2 generation by high resolution melting (HRM) point analysis for the presence of sequence polymorphisms at the target site. The CRISPR/Cas9 transgene was out segregated in the T3 generation and plants carrying the *smg7-7* allele in a heterozygous constitution were identified by HRM analysis and sequencing. Plants were characterized in the T4 and T5 generations. The complementation constructs for functional significance of the 14-3-3 domain of SMG7 were as follows: 6.9 kb of SMG7 genomic sequence including 1.5 kb promoter and 3′UTR was PCR amplified using primers SMG7::SMG7-SbfI and SMG7::SMG7-SacI (Supplementary Table S1) and cloned into pJET1.2/blunt Cloning Vector (Thermo Fisher Scientific). For site directed mutagenesis, a smaller fragment of SMG7 gene was amplified with the primers F-SMG7-SpeI and R-SMG7-HpaI and the PCR product was subcloned in the pJET1.2/blunt Cloning Vector. Site directed mutagenesis of K77E and R185E was done in two rounds of PCR, first with primers F-SMG7-K77E and R-SMG7-K77E, and second with primers F-SMG7-R185E and R-SMG7-R185E. The mutated fragment was subcloned back into SpeI/HpaI of the SMG7 gene construct. Wild type and mutated versions of the SMG7 gene were subcloned into the PstI/SacI sites of the binary vector pCBK06 (Riha et al., 2002) and resulting constructs were transformed into *Agrobacterium tumefaciens* GV3101. Complementation lines were generated by transforming Arabidopsis heterozygous for the *smg7-1* allele by floral dip technique.

### Fertility Assays

Pollen viability was determined by Alexander staining (Alexander, 1969). Meiosis in pollen mother cells was analyzed by staining whole anthers with DAPI followed by confocal microscopy according to (Brownfield et al., 2015).

### Gene Expression Analysis

Total RNA was isolated from plant leaf tissue using RNA Blue (Top-Bio) following the manufacturer’s protocol. Quality and quantity of isolated RNA were checked on a 1.2% denaturing agarose gel and with the NanoDrop 2000C spectrophotometer (Thermo Fisher Scientific), respectively. For each sample, 10 μg of total RNA was treated with DNase I (Roche) to remove genomic DNA and 2 μg DNA-free RNA was reverse transcribed using Superscript IV (Invitrogen) following the manufacturer’s protocol. For qRT-PCR reactions, cDNA was diluted two times and all reactions were carried out using EvaGreen dye (Biotium), GoTaq polymerase (Promega, United States), and gene-specific primers on a LightCycler 96 (Roche). Expression was normalized to At2G28390 and relative fold change was calculated using the delta-delta Ct method (Livak and Schmittgen, 2001). All qRT-PCR reactions were performed on at least three biological replicates and 2–3 technical replicates. The sequence of all primers used in this study are listed in Supplementary Table S1.

### Protein Alignment

We downloaded the full-length protein sequences of SMG7 and SMG7L from 13 plant species (*Physcomitrella patens*, *Selaginella moellendorffii*, *Amborella trichopoda*, *Medicago truncatula*, *Arabidopsis thaliana*, *Brassica oleracea*, *Cucumis sativus*, *Nicotiana attenuata*, *Helianthus annuus*, *Oryza sativa*, *Zea mays*, *Sorghum bicolor*, *Hordeum vulgare*) from Ensembl Plants (release 38) and of human (*Homo sapiens*) from Ensembl (release 91). The following sequences were used for alignment: *Hs*SMG7 (ENSP00000425133), *Pp*SMG7a (PP1S80_14V6.1), *Pp*SMG7b (PP1S311_73V6.1), *Sm*SMG7a (EFJ27061), *Sm*SMG7b (EFJ21470), *Amt*SMG7 (ERN18017), *Mt*SMG7a (KEH28378), *Mt*SMG7b (KEH16467), *At*SMG7 (AT5G19400.1), *At*SMG7L (AT1G28260.1), *Bo*SMG7a (Bo9g153800.1), *Bo*SMG7b (Bo2g018020.1), *Bo*SMG7La (Bo5g054690.1), *Bo*SMG7Lb (Bo3g143280.1), *Cs*SMG7 (KGN66550), *Cs*SMG7L (KGN64688), *Na*SMG7 (OIS97991), *Na*SMG7L (OIT28005), *Ha*SMG7a (OTG27135), *Ha*SMG7b (OTG30173), *Ha*SMG7L (OTF97490), *Os*SMG7 (Os08t0305300-01), *Zm*SMG7a (Zm00001d019920_P002), *Zm*SMG7b (Zm00001d005502_P002), *Sb*SMG7 (KXG35214), *Hv*SMG7a (HORVU5Hr1G050800.5), *Hv*SMG7b (HORVU0Hr1G029520.1). Protein alignment was performed with the MegAlign Pro function of DNASTAR Navigator (v12.2.0.80) using the MUSCLE (Multiple Sequence Comparison by Log-Expectation) method. The multiple sequence alignment (MSA) for selected sequence features of the SMG7 protein across the plant kingdom was used to generate a sequence logo using the online version of WebLogo3 (Crooks et al., 2004). SMG7 specific regions were identified by visually inspecting multiple sequence alignment of plant SMG7/SMG7L proteins for regions that are conserved in SMG7 paralogs of monocots and dicots, but diverged in SMG7L.

## Results

### Full Inactivation of Arabidopsis SMG7 Does Not Result in Embryonic Lethality

**FIGURE 1:**
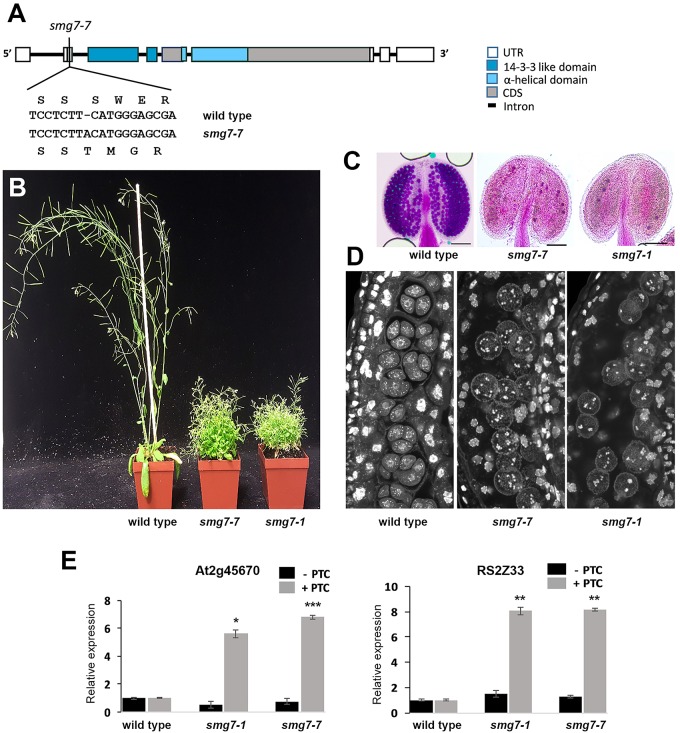
Comparison of Arabidopsis *smg7-7* and *smg7-1* mutants. **(A)** Schematic diagram of the SMG7 gene with the position of the *smg7-7* mutation indicated. **(B)** Approximately 6-week-old wild type and mutant plants. **(C)** Anthers with viable pollen visualized by Alexander staining. **(D)** Developing pollen mother cells within an anther stained by DAPI. Tetrads are apparent in wild type, while late *smg7* meiocytes contain randomly distributed chromatids. **(E)** Effect of *smg7* alleles on the relative abundance of two alternatively spliced variants of the same transcript as determined by real time RT-PCR. Error bars represent SEM of three biological replicas. Asterisks indicate statistical significance of difference from wild type (^∗^*P* < 0.5, ^∗∗^*P* < 0.01, ^∗∗∗^*P* < 0.001, two-tailed *t*-test).

In our attempts to genetically complement the *smg7-5* mutation with a functional *SMG7* gene, we failed to recover plants homozygous for the *smg7-5* allele. This led us to ask whether the embryonic lethality observed in the *smg7-5* line (Riehs et al., 2008) is indeed linked to the T-DNA insertion in the SMG7 gene. To clarify this issue, we used CRISPR/Cas9 targeted mutagenesis to generate another mutation that disrupts the conserved 14-3-3 domain. We targeted the Cas9 nuclease to the second exon of the SMG7 gene and identified an allele that contains a frame shift mutation 13 amino acids downstream of the ATG codon and hence likely represents a full loss of function allele that we call *smg7-7* (Figure 1A). We were able to recover viable plants homozygous for the *smg7-7* allele that phenotypically resembled the growth-retarded *smg7-1* mutants (Figure 1B). These plants were sterile and did not produce any pollen (Figure 1C). As the infertility of *smg7-1* was caused by arrest of meiotic progression in anaphase of the second meiotic division (Riehs et al., 2008), we performed cytogenetic analysis of pollen mother cells in anthers of *smg7-7* mutants. This revealed meiocytes with irregularly distributed chromosomes, which is typical for this anaphase II arrest (Figure 1D). To test the effect of *smg7-7* on NMD, we used an assay that quantifies two alternatively spliced variants of the same transcript, one of which contains a PTC (Gloggnitzer et al., 2014; Figure 1E). We observed comparable upregulation of two different PTC containing transcripts in *smg7-1* and *smg7-7*, suggesting that NMD is impaired to the similar extent in both alleles. Thus, *smg7-1* and *smg7-7* lead to the same effect on NMD and meiosis, demonstrating that the *smg7-1* allele used in our previous studies likely represents a full loss of function mutation and that inactivation of SMG7 is not lethal, in contrast to the conclusion previously reported (Riehs et al., 2008).

### Arabidopsis SMG7L Does Not Compensate for the Loss of SMG7

**FIGURE 2:**
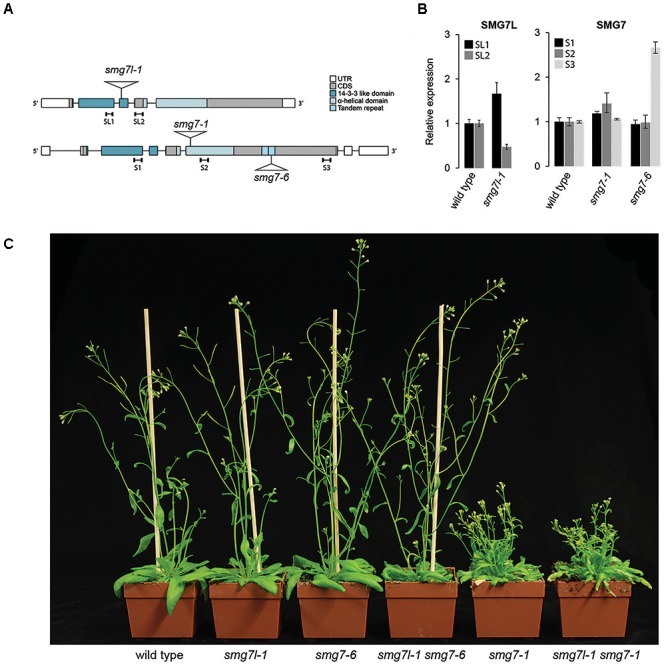
Double mutants of *SMG7* and *SMG7L*. **(A)** Diagram of *SMG7* and *SMG7L* genes with positions of T-DNA insertions and functional domains indicated. **(B)** qRT-PCR analysis of SMG7L and SMG7 transcripts. Positions of the regions amplified by PCR are indicated in **(A)**. Error bars represent SEM of three biological replicas. **(C)** Five week-old wild type and mutant plants.

Our observation that complete loss of SMG7 does not cause embryonic lethality implies that the consequence of SMG7 inactivation is milder than that of UPF1. This led us to ask whether SMG7L can partially compensate for the loss of SMG7 in NMD. To address this question, we generated plants with mutations in both genes. We combined *smg7l-1*, which carries a T-DNA insertion in the N-terminal 14-3-3 like domain, with either the severe *smg7-1*, or the hypomorphic *smg7-6* (Figure 2A). *smg7-6* contains a T-DNA insertion in the C-terminus and was reported to be proficient for NMD but partially impaired in meiosis (Riehs-Kearnan et al., 2012). Quantitative RT-PCR analysis showed that mRNA encoding the N-terminal portion of the SMG7 protein is expressed at the same level in *smg7-6* as in wild type plants, suggesting the production of a truncated, partially functional protein (Figure 2B). Plants deficient for SMG7L did not show any obvious phenotype and were indistinguishable from wild type plants (Figure 2C).

**FIGURE 3:**
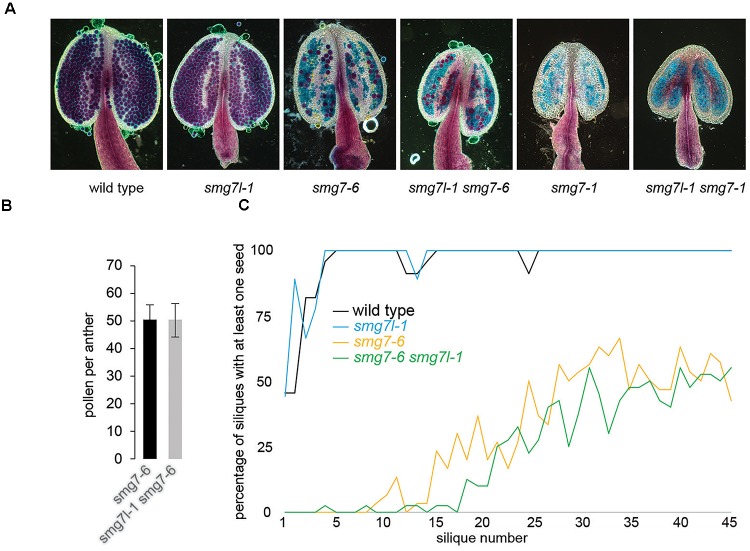
Fertility in plants deficient for *SMG7L* and *SMG7*. **(A)** Anthers with viable pollen visualized by Alexander staining. **(B)** Pollen count per anther in *smg7-6* and *smg7l-1 smg7-6*. Error bars represent standard deviation from 35 anthers collected from 10 floral buds. **(C)** Proportion of siliques at a given position on the main stem producing at least one seed (1 represents the bottom-most silique). 9–20 plants were counted for each genotype.

**FIGURE 4:**
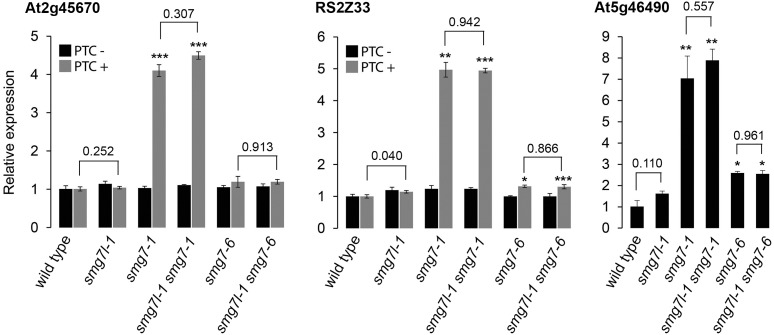
Relative abundance of transcripts known to be targeted by NMD (Gloggnitzer et al., 2014). Error bars represent SEM from three biological replicas. Asterisks indicate statistical significance of difference from wild type (^∗^*P* < 0.5, ^∗∗^*P* < 0.01, ^∗∗∗^*P* < 0.001); numbers above horizontal bars represent significance of difference between SMG7L deficient plants and their respective controls.

We anticipated that if SMG7L acts redundantly with SMG7, it should exacerbate the phenotypes of both *smg7* alleles. However, neither *smg7l-1 smg7-1* nor *smg7l-1 smg7-6* double mutants exhibited any difference from the respective *smg7* single mutants with regards to growth defects and fertility (Figures 2C, 3). While *smg7-1* is infertile, *smg7-6* plants produce a reduced amount of viable pollen and are partially fertile (Figures 3A,B). Usually, the first 15–20 flowers on the main inflorescence bolt are infertile, while later flowers produce viable seeds (Figure 3C). Quantification of pollen and seed production did not reveal any difference between *smg7-6* and *smg7l-1 smg7-6*, arguing that SMG7L does not compensate for the meiotic function of SMG7. We assessed the efficiency of NMD by quantifying three different endogenous transcripts known to be degraded by NMD (Figure 4; Gloggnitzer et al., 2014). The *smg7-1* null mutation displayed increased amounts of all transcripts targeted by NMD (Figure 4). Although we have previously reported that the hypomorphic *smg7-6* allele is NMD proficient (Riehs-Kearnan et al., 2012), we observed a slight but reproducible increase of the three NMD-targeted transcripts in this study (Figure 4) suggesting a very mild NMD defect. Importantly, the *smg7l-1 smg7-1* and *smg7l-1 smg7-6* double mutants did not show any additional increase in NMD-regulated transcripts. Together, these data allow the conclusion that SMG7L does not act redundantly with SMG7 either in meiosis or in NMD.

### The N-Terminal 14-3-3 Domain of SMG7 Is Important for Meiosis

**FIGURE 5:**
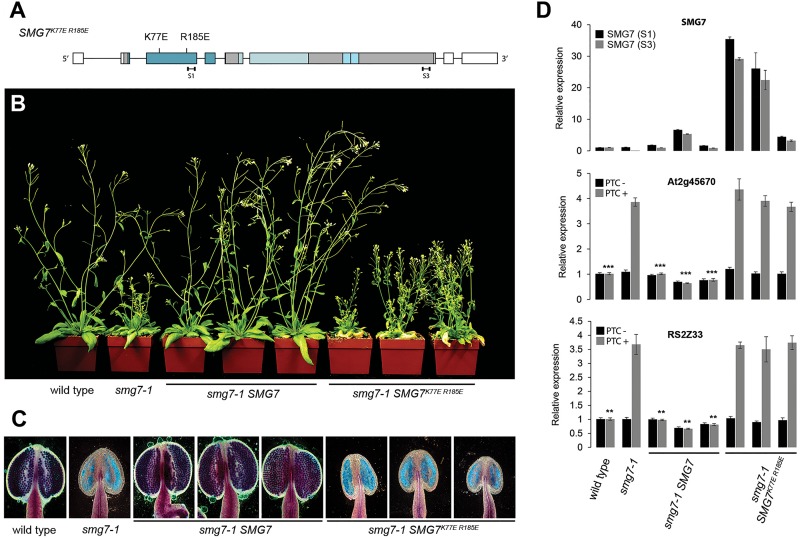
Complementation of *smg7-1* with *SMG7^K77E^^*R185E*^*. **(A)** Schematic diagram of the SMG7 gene with the K77E and R185E mutations indicated. **(B)** Five week-old T2 *smg7-1* plants complemented with either the wild type or *SMG7^K77E^^*R185E*^* construct. **(C)** Anthers assayed by Alexander staining. **(D)** Relative abundance of SMG7 and two NMD reporter genes determined by real time RT-PCR. Two sets of primers were used for quantification of SMG7 mRNA (S1, S3) and their position is indicated in panel **A**. Error bars represent SEM from three biological replicas. Asterisks indicate statistical significance of difference from *smg7-1* mutants (^∗^*P* < 0.5, ^∗∗^*P* < 0.01, ^∗∗∗^*P* < 0.001, two-tailed *t*-test). Plants derived from three independent transformants are shown in each category in **(B–D)**.

The genetic separation of NMD and meiotic functions in *smg7-6* mutants together with the fact that UPF1 and UPF3 deficient plants lack a meiotic phenotype (Riehs-Kearnan et al., 2012) indicate that SMG7 acts in meiosis through a different mechanism than in NMD. Thus, we wanted to know whether the conserved 14-3-3 domain that mediates interaction with phosphorylated UPF1 in NMD is also required for meiosis. We complemented the *smg7-1* with a copy of the Arabidopsis SMG7 gene carrying mutations in the conserved K77 and R185 residues that form the phosphoserine-binding pocket and are required for NMD in tobacco (Figure 5A; Fukuhara et al., 2005; Merai et al., 2012). We transformed plants heterozygous for *smg7-1* with constructs carrying either wild type *SMG7* or the mutant *SMG7^K77E^^*R185E*^* and identified transformants homozygous for *smg7-1* in segregating T2 populations. Three independent lines were analyzed for each construct. While the wild type construct readily complemented the growth phenotype, fertility, and pollen production, *SMG7^K77E^* ^*R185E*^ plants were indistinguishable from *smg7-1* mutants despite higher level of SMG7 mRNA expression (Figures 5B,C). As expected, the *SMG7^K77E^^*R185E*^* plants were deficient in NMD, but they also failed to produce any pollen (Figures 5C,D). This indicates that the phosphoserine-binding activity mediated by the conserved N-terminal 14-3-3 motif is important not only for NMD, but also for the meiotic function of SMG7.

### The Meiotic Function of SMG7 May Depend on Conserved Motifs in Its C-Terminus

Studies in grapevine using virus induced gene silencing (Benkovics et al., 2011) together with our phenotypic characterization of Arabidopsis mutants demonstrated that SMG7 and SMG7L paralogs functionally diverged in dicots. While SMG7 retained its function in NMD, SMG7L apparently acquired a novel function(s) unrelated to NMD and meiosis. We hypothesized that this neofunctionalization would be accompanied by amino acid sequence divergence in regions exclusively important for NMD. According to this hypothesis, SMG7 should have retained motifs involved in NMD whereas some of these motifs may have been lost in SMG7L.

**FIGURE 6:**
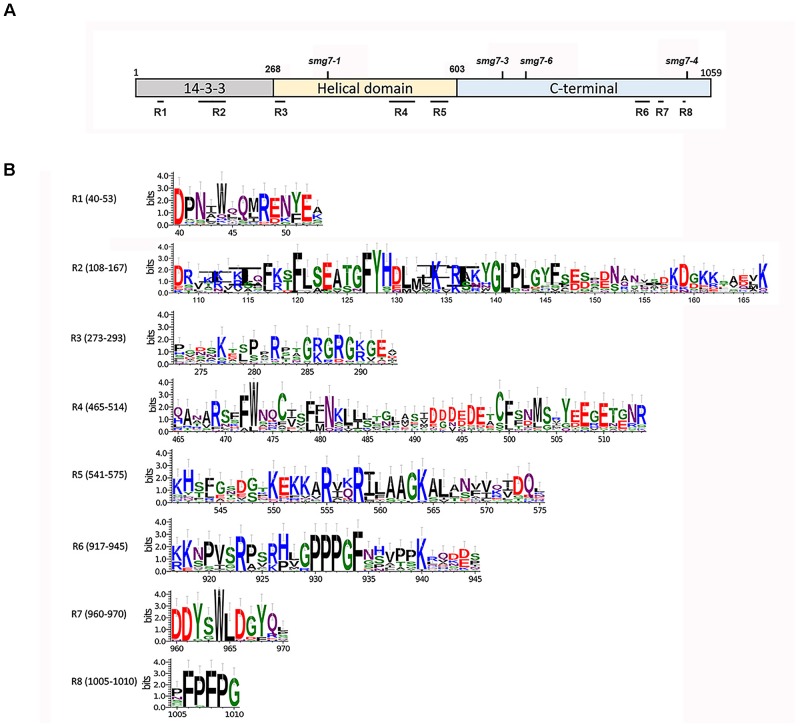
Amino acid sequence motifs specific for the Arabidopsis SMG7 protein. **(A)** Schematic representation of the SMG7 protein and of its domains. Amino acid coordinates for domain boundaries, positions of SMG7-specific regions, and disruptions of SMG7 by T-DNA insertions are indicated. **(B)** Consensus sequences of individual SMG7-specific regions assembled from alignment of proteins from 13 plant species (Supplementary Figure S1). The *x*-axis represent the position in the *A. thaliana* SMG7 protein sequence. The *y*-axis labels represent the information content of the position in sequence (in bits).

We performed a sequence comparison of multiple plant SMG7 and SMG7L proteins to identify regions that are conserved across the plant kingdom but which diverged during the evolution of SMG7L in dicots. Five such regions were identified in the evolutionary conserved N-terminal half of SMG7, and three additional SMG7-specific motifs were found at the C-terminus (Figure 6). While all 6 amino acids residues that form the phosphoserine-binding pocket of the 14-3-3 domain are retained in both SMG7 and SMG7L, two regions in the 14-3-3 domain diverged between these paralogs. When mapped over the structure of human SMG7 (Fukuhara et al., 2005), the plant SMG7-specific regions 1 and 2 correspond to alpha helices α2 and α4 with the connecting loop to α5, respectively. The α2 and α4 helices are aligned on the convex surface of the 14-3-3 domain, and α4 together with the extended loop was reported to form an interaction interface with SMG5 (Jonas et al., 2013). Thus, it is likely that these regions form a protein interaction surface and their divergence in SMG7L reflects altered protein-binding specificity. The SMG7-specific region 3 is located in the loop that connects the 14-3-3 domain with the helical domain and is larger in SMG7 than in SMG7L paralogs (Figure 6). The SMG7-specific regions 4 and 5 span helices α16 and α18 at the end of the helical domain. The C-terminal portion of SMG7 is generally less conserved than the N-terminus. Nevertheless, we found three motifs at the C-terminus of SMG7 proteins (motifs 6, 7, and 8) that are shared among monocots, dicots, and moss, but were lost in dicot SMG7L (Figure 6).

## Discussion

SMG7 is an ancient phosphoserine-binding protein that is present in most eukaryotes. Its primary function is linked to NMD as its physical and functional interactions with UPF1 are conserved from plants to animals (Unterholzner and Izaurralde, 2004; Kerenyi et al., 2013). Nevertheless, proteins of the SMG7 family have acquired additional functions during evolution. The best known example is Est1p in budding yeast that recruits telomerase to chromosome ends via binding to phosphorylated Cdc13 (Li et al., 2009; Chen et al., 2018). Hence, SMG7 acts as an adaptor protein that can recruit different molecular machines to the sites of their action in a phosphorylation dependent manner. Arabidopsis SMG7 is involved in at least two independent molecular processes, NMD and meiotic progression. Here we show that its N-terminal 14-3-3 domain is required for both NMD and meiosis. While UPF1 is the substrate that defines the role of SMG7 in NMD (Kerenyi et al., 2013), the meiotic function is likely mediated by another binding protein as Arabidopsis deficient for UPF1 does not exhibit the meiotic defects described for *smg7* mutants (Riehs-Kearnan et al., 2012).

Proteins of the SMG7 gene family underwent independent multiplications during evolution that were accompanied by functional diversification. In vertebrates SMG5, SMG6, and SMG7 paralogs play distinct roles in NMD that define two separate RNA degradation pathways. Multiple copies of SMG7 have also independently arisen in plants including maize, poplar, grapevine, or the moss *P. patens* (Benkovics et al., 2011; Li et al., 2015). In the majority of cases, these are relatively recent species-specific duplications that only occasionally span larger phylogenetic units. SMG7L represents the most ancient plant duplication, originating at the root of dicots (Riehs et al., 2008). Here we demonstrate that SMG7L does not act redundantly with SMG7 in either NMD or meiosis implying that it evolved a novel, yet unknown function. Domain swapping experiments indicated that the N-terminal portion of SMG7L retained its capability to bind UPF1 and complement NMD, whereas the C-terminus lost its capability to trigger RNA degradation in a tethering assay (Benkovics et al., 2011). Nevertheless, it is unlikely that UPF1 is a physiological substrate of SMG7L; sequence divergence in the loops and helices that form the surface interfaces of the conserved N-terminal domains rather suggest that SMG7L binds other proteins.

Phylogenetic comparison of SMG7 and SMG7L protein sequences revealed several regions that were conserved in SMG7, but diverged in SMG7L. Positions of the SMG7-specific motifs in respect to mutations that have been functionally characterized (Figure 6A) may provide clues on their function. Arabidopsis *smg7-1* and *smg7-3* represent T-DNA insertions that truncate SMG7 at amino acid positions 355 and 675, and both alleles are deficient in NMD and meiosis (Riehs et al., 2008; Riehs-Kearnan et al., 2012). The *smg7-6* disruption is only 43 amino acids downstream from *smg7-3* at position 718 yet is only very mildly impaired in NMD, implying a functional significance of this region. Nevertheless, the region between amino acids 675 and 718 is highly divergent among plant SMG7 homologs (Supplementary Figure S1), which speaks against a specific role for this region in NMD. Since *smg7-3* is disrupted shortly after a motif conserved in both SMG7 and SMG7L paralogs, this mutation may affect the proper folding or stability of the truncated protein. Experiments using a transient expression in tobacco showed that the Arabidopsis SMG7 C-terminal domain is required for NMD and that tethering a C-terminal fragment (amino acids 517–1059) to RNA is sufficient to trigger its degradation (Benkovics et al., 2011). Thus, the region responsible for RNA degradation must be located between amino acids 517 and 718, which overlaps with the SMG7-specific region 5 (Figure 6). The impaired fertility of *smg7-6* indicates a requirement for the SMG7 C-terminus in meiosis. It is likely that the meiotic function of SMG7 is mediated additionally through conserved regions 6, 7, and 8. In support of this notion, Arabidopsis *smg7-4* which carry a T-DNA disruption at amino acid position 1013 immediately after the SMG7-specific region 8 are fully fertile (Riehs-Kearnan et al., 2012).

Whether the conserved function of these motifs is specifically linked to reproduction, or whether they underlie a general molecular function, for which deficiency in Arabidopsis is manifested as a meiotic defect, remains to be seen.

## Author Contributions

CC, NS, KR, and OMS designed the experiments. CC performed the experiments shown in Figures 1–5. NS performed the experiments shown in Figures 1, 6. AW generated the *smg7-7* mutant plants. KR and NS wrote the manuscript.

## Conflict of Interest Statement

The authors declare that the research was conducted in the absence of any commercial or financial relationships that could be construed as a potential conflict of interest.

## Supplementary Material

FIGURE S1 Sequence alignment of the following SMG7 proteins: *Hs*SMG7 (ENSP00000425133), *PpS*MG7a (PP1S80_14V6.1), *Pp*SMG7b (PP1S311_73V6.1), *Sm*SMG7a (EFJ27061), *Sm*SMG7b (EFJ21470), *Amt*SMG7 (ERN18017), *Mt*SMG7a (KEH28378), *Mt*SMG7b (KEH16467), *At*SMG7 (AT5G19400.1), *At*SMG7L (AT1G28260.1), *Bo*SMG7a (Bo9g153800.1), *Bo*SMG7b (Bo2g018020.1), *Bo*SMG7La (Bo5g054690.1), *Bo*SMG7Lb (Bo3g143280.1), *Cs*SMG7 (KGN66550), *Cs*SMG7L (KGN64688), *Na*SMG7 (OIS97991), *Na*SMG7L (OIT28005), *Ha*SMG7a (OTG27135), *Ha*SMG7b (OTG30173), *Ha*SMG7L (OTF97490), *Os*SMG7 (Os08t0305300-01), *Zm*SMG7a (Zm00001d019920_P002), *Zm*SMG7b (Zm00001d005502_P002), *Sb*SMG7 (KXG35214), *Hv*SMG7a (HORVU5Hr1G050800.5), *Hv*SMG7b (HORVU0Hr1G029520.1).

TABLE S1 Primers used in this study.
