# Supplementary figures and images for: Functional Characterization of SMG7 Paralogs in Arabidopsis thaliana
Source: Front Plant Sci. 2018 Nov 6;9:1602. doi: 10.3389/fpls.2018.01602 (PMC6232500; doi:10.3389/fpls.2018.01602)

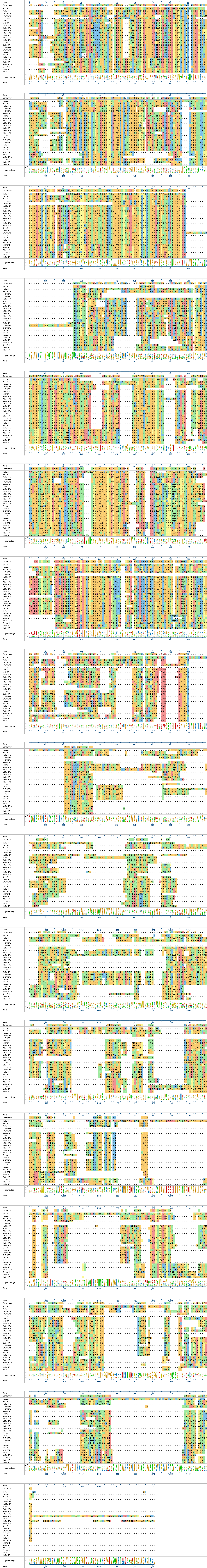

Supplement: FIGURE S1 — Sequence alignment of the following SMG7 proteins: HsSMG7 (ENSP00000425133), PpSMG7a (PP1S80_14V6.1), PpSMG7b (PP1S311_73V6.1), SmSMG7a (EFJ27061), SmSMG7b (EFJ21470), AmtSMG7 (ERN18017), MtSMG7a (KEH28378), MtSMG7b (KEH16467), AtSMG7 (AT5G19400.1), AtSMG7L (AT1G28260.1), BoSMG7a (Bo9g153800.1), BoSMG7b (Bo2g018020.1), BoSMG7La (Bo5g054690.1), BoSMG7Lb (Bo3g143280.1), CsSMG7 (KGN66550), CsSMG7L (KGN64688), NaSMG7 (OIS97991), NaSMG7L (OIT28005), HaSMG7a (OTG27135), HaSMG7b (OTG30173), HaSMG7L (OTF97490), OsSMG7 (Os08t0305300-01), ZmSMG7a (Zm00001d019920_P002), ZmSMG7b (Zm00001d005502_P002), SbSMG7 (KXG35214), HvSMG7a (HORVU5Hr1G050800.5), HvSMG7b (HORVU0Hr1G029520.1). [file Image_1.PNG]
